# Efficient Extraction and Separation of Scandium from Scandium-Bearing Solid Waste and Acid by Synergistically Leaching Followed by Solvent Extraction

**DOI:** 10.3390/molecules29194766

**Published:** 2024-10-09

**Authors:** Wen Cao, Jinmao Hua, Xi Jin, Minyu He, Yuntao Xin, Weizao Liu

**Affiliations:** 1 College of Materials Science and Engineering, Chongqing University, Chongqing 400044, China; 2 College of Chemistry and Chemical Engineering, Central South University, Changsha 410083, China

**Keywords:** solvent extraction, scandium, solid waste, waste acid, iron removal

## Abstract

The solid waste and waste acid generated during the production of titanium dioxide contain considerable amount of scandium, which are valuable secondary resources. In this study, the titanium dioxide waste acid was used to leach the scandium-containing solid waste, and the leached solution was pretreated for iron removal by reduction-crystallization process. After that, scandium was recovered from the leached solution by using the P204-TBP co-extraction system. The process parameters were investigated systematically. The results showed that iron powder reduction-crystallization for iron removal at molar ratio of Fe:Fe^3+^ = 0.25 was most suitable for subsequent extraction, and the scandium extraction efficiency could reach 100% using 15% P204-5% TBP at 25 °C with A/O = 8. This study provided a novel process for treating scandium-bearing solid waste with scandium-bearing waste acid, showing great potential for industrial application.

## 1. Introduction

Scandium is one of the rare earth elements, which is regarded as a strategic resource due to its irreplaceable applications in a series of modern fields, such as new energy materials, modern information technology, aerospace, and national defense and military [1,2]. However, the deposits with high content of scandium are scarce, and scandium is usually coexisted with other ores, such as lateritic ores [3,4], vanadium-titanium magnetite ores [5], rare earth ores [6,7]. As the primary elements are extracted from these ores, scandium usually ends up in the resulting waste water and slag [8]. Therefore, secondary resources become the main source for scandium extraction, such as titanium dioxide waste acid [9,10], red mud [11,12,13], tungsten slag [14], bauxite slag [15,16,17], rare earth slag [18], fly ash [19].

The waste acid discharged from titanium dioxide production is one of the resources of scandium, and 6–8 tonnes of waste acid would be generated for producing per tonne of TiO_2_. In 2023, the output of titanium dioxide by sulfuric acid process in China was 4.16 million tonnes, and the by-production of waste acid amounted to 20.8–27.75 million tonnes. The titanium dioxide waste acid (TWWA) mainly contains Fe, Ti, Al, Mg, Ca, and a very low concentration of Sc (5–20 mg/L) with the acidity of around 20% [20]. Therefore, TWWA is considered as an important source of scandium. Meanwhile, the wastewater generated in the titanium production process also contains a small amount of scandium, which is generally neutralized by lime to form a scandium-containing slag. The Sc content in the scandium-containing waste slag is also low, which makes it difficult to be utilized directly.

To recover scandium from scandium-containing resources, the scandium in the secondary resources is usually extracted into liquid phase by acid leaching or sulfuric acid roasting-water leaching. And then, the scandium is separated from the leached solution. There are many methods for the separation and purification of scandium, and solvent extraction is the most economical and effective method due to its simple process, convenient operation, low consumption and high extraction efficiency [1,21]. The commonly used extractants are Di-(2-ethylhexyl) phosphoric acid (P204), 2-ethylhexyl phosphonic acid mono- 2-ethylhexyl ester (P507) [22], trialkyl phosphine oxide (TRPO) [23], primary amine N1923 [20], Cyanex 572 [24],trialkyl phosphine oxides (Cyanex 923) [25]. Among them, P204 is widely used for the separation and purification of scandium from titanium dioxide waste acid due to its high extraction efficiency and lower price [21]. However, it is difficult to obtain high purity scandium due to the fact that titanium dioxide waste acid contains elements that can be co-extracted, such as Fe(III), Ti and Zr [26]. Meanwhile, Fe(III) presented in the waste acid is easily co-extracted by P204 at low pH during the extraction process, which competes with Sc, leading to a decrease in the extraction efficiency of Sc [27]. What’s worse, Fe(III) would lead to the generation of the third phase in the extraction process, resulting in the separation of oil phase from liquid phase becomes difficult [28]. Therefore, the efficient extraction of scandium without generating a third phase needs further investigation.

Wang et al. [28] carried out extraction experiments on the acid--leached solution of red mud using P204 and found that the extraction efficiency of scandium could reach more than 99.5%. However, the emulsification phenomenon could not be avoided when a single extractant was used. The emulsification phenomenon could be mitigated when Tri-butyl phosphate (TBP) was used as a phase modifier, but the extraction efficiency was decreased. Chen et al. [9] recovered scandium from titanium dioxide waste acid using 10% P204-5% TBP, and the extraction efficiency could reach 99% under the optimal extraction conditions of A/O = 30, 30 min, 25 °C. Zhang et al. [29] recovered scandium from titanium dioxide waste acid using 8% P204-2% TBP, and the extraction efficiency could reach 99.98%. However, to completely solve the emulsification problem, it is still necessary to remove iron and reduce the content of iron ions (Fe(III)) before solvent extraction.

Since titanium dioxide waste acid contains a small amount of Sc, while the scandium-containing waste slag also contains a small amount of scandium, they are valuable secondary resources. In this study, titanium dioxide waste acid was used to leach scandium-containing waste slag to enrich scandium, and the effects of temperature, liquid-solid ratio and time on leaching were investigated to determine the optimal leaching conditions. The leached solution was subjected to iron removal. Four methods, namely potassium ferricyanide method, yellow potassium ferrovanadium method, ferric amine sulfate crystallization method and iron powder reduction-crystallization method, were compared to determine the best iron removal method. Finally, the synergistic extraction system of P204-TBP was used to extract the Fe(III)-depleted solution, and the effects of iron removal amount, P204 content, A/O ratio and temperature were studied to determine the optimal extraction conditions. The conceptual flowchart of scandium enrichment from waste acid and slag was proposed.

## 2. Results and Discussion

### 2.1. Characterization

**Figure 1:**
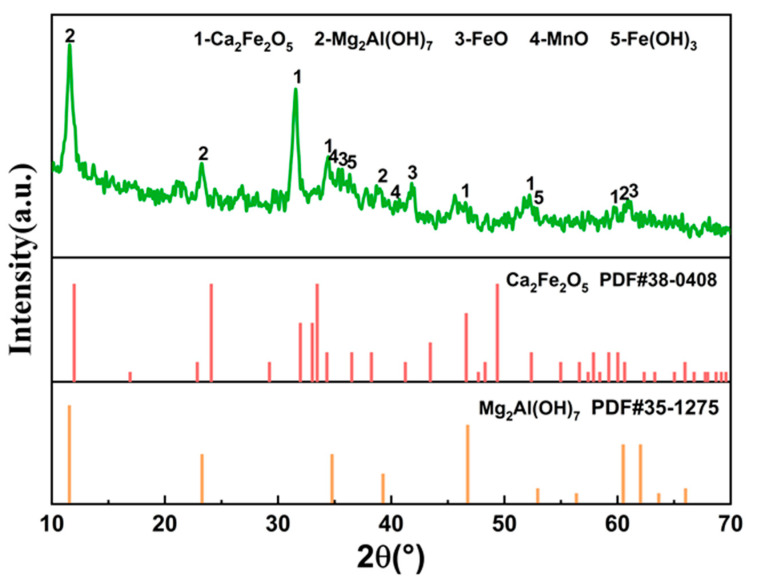
X-ray diffraction (XRD) test results of waste slag.

The waste slag was characterized using X-ray fluorescence (XRF) and X-ray diffraction (XRD), and the results are shown in Table 1 and Figure 1. As seen from Table 1, there are many impurity elements in the waste slag, in which the content of Fe reached 47.2% and was much larger than the other elements. The content of Sc was not detected due to its low content, while the presence of Ti, Al, Zr and other elements in the slag may affect the subsequent extraction and separation of Sc. As can be seen from Figure 1, the waste slag was mainly composed of hydroxides and oxides, but Sc-bearing phases were not detected due to the low content. The metal ions contents of the slag and the titanium dioxide waste acid were tested by ICP-OES, and the results are shown in Table 2. The results also confirmed that the highest content of Fe elements in the slag and waste acid The content of Sc was very small with 0.0017% in the slag and 15.1 mg/L in the waste acid, respectively.

### 2.2. Leaching for Scandium Enrichment

#### 2.2.1. Effect of Leaching Temperature

**Figure 2:**
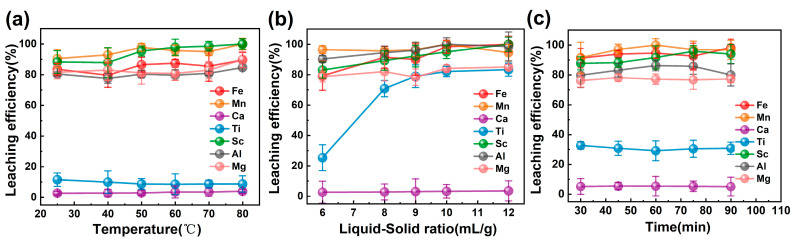
(**a**) Effect of leaching temperature (L/S = 10 mL/g, 1 h), (**b**) Effect of liquid-solid ratio (1 h, 25 °C), (**c**) Effect of leaching time (L/S = 6 mL/g, 25 °C).

The effect of leaching temperature on the leaching efficiency is shown in Figure 2a. The leaching efficiency of Sc showed an increasing trend from 88.21% to 100% with the increase in temperature, while the leaching efficiency of elements such as Fe, Mg, Mn and Al also increased with the increase in temperature. The leaching of elements such as Ca and Ti was almost unchanged with the temperature. Therefore, considering the leaching effect of each impurity element and economic benefits, the subsequent experimental study was selected to be carried out at room temperature.

#### 2.2.2. Effect of Liquid-Solid Ratio

The effect of liquid-solid ratio (L/S) is shown in Figure 2b. The leaching efficiency of Sc increased from 83.02% to 100% with the increase in liquid-solid ratio, and the leaching efficiency of Fe and Ti also showed an increasing trend. Notably, the leaching efficiency of Ti increased sharply when the liquid-solid ratio went from 6 to 8. This may be due to the fact that the increase in the titanium dioxide waste acid provided the capacity for the leaching of Ti from the slag. The leaching efficiencies of Mg, Mn, Ca, Al and other elements did not change much. The liquid-solid ratio of 6 was selected for the subsequent experiments due to the sharp increase in Ti leached.

#### 2.2.3. Effect of Leaching Time

The effect of leaching time is shown in Figure 2c. Leaching time had less effect on the leaching of each element. When the leaching time reached 60 min, Fe, Mg, Mn, Ca, Al and other impurity elements reached their highest values. When the leaching time was 30 min, the leaching efficiency of Sc is 87.72%, and the leaching efficiency of impurity elements is lower than that in 60 min, which can reduce the influence of the subsequent extraction process. Thus, the leaching time of 30 min was determined to be more appropriate.

In summary, according to the experimental results, the liquid-to-solid ratio had the greatest impact on the leaching efficiency of each element, followed by temperature, while the effect of time on leaching efficiency was minimal. This may be due to the fact that a significant amount of each element can be leached within 30 min; therefore, extending the leaching time beyond this point did not lead to significant changes in the leaching outcomes of the elements. The optimum leaching conditions were selected as room temperature, liquid-solid ratio L/S = 6 mL/g and leached for 30 min. The changes in the content of metal elements in the solution before and after leaching can be seen in Table 3. Under optimal conditions, the content of Sc in the solution was enriched from 15.1 mg/L to 38.1 mg/L, but the amount of Fe element was also increased accordingly. Therefore, the subsequent iron removal study is required.

### 2.3. Pre-Iron Removal for Leached Solution

**Figure 3:**
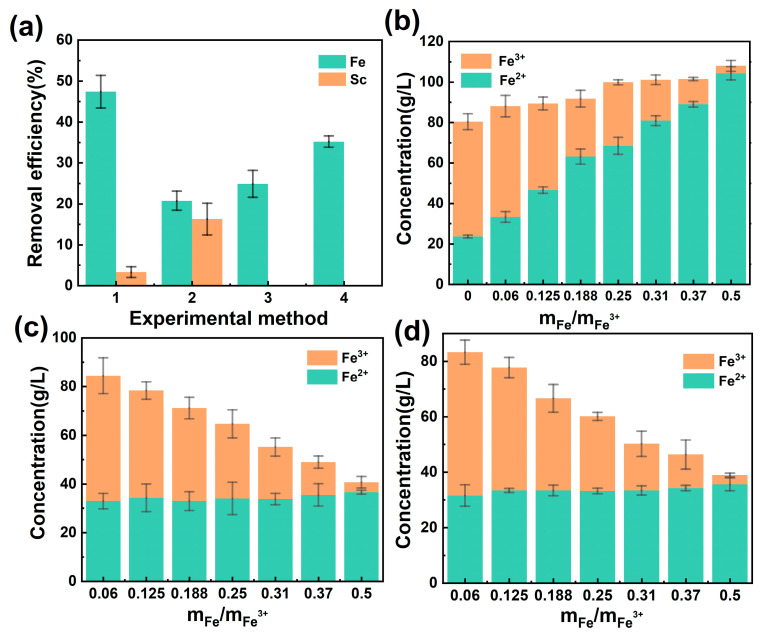
(**a**) Comparison of different methods of iron removal (1-potassium ferricyanide method, 2-jarosite process, 3-ammonium iron sulfate crystallization, 4-powdered iron reduction and crystallization); (**b**–**d**) Changes in Fe^3+^ vs. Fe^2+^ (after iron powder reduction, after crystallization, after solvent extraction).

Since the content of Fe element in the leached solution was much higher than that of Sc, in which the large amount of Fe^3+^ would reduce the extraction efficiency of scandium, and easy to form the third phase during the extraction process. Thus, it was necessary to remove iron (Fe(III)) from the leached solution before solvent extraction. Four commonly used iron removal methods were performed. The efficiency of the four methods for iron removal is depicted in Figure 3a. As can be seen from this Figure, the iron removal effect of potassium ferrocyanide method can reach 47.4%, while the loss of scandium is very low, only 3.29%. However, the method was prone to the formation of colloid, leading to the difficulty of pumping and filtration during the experiment process. Compared with the iron removal method of potassium ferricyanide, the iron removal efficiencies of the other three methods were lower: 20.76%, 24.88% and 35.23%, respectively. However, the loss ratio of scandium in the jarosite process reached 16.29%, which may be the result of entrapping scandium along with the precipitation. There was no scandium loss in the ammonium iron sulfate crystallization method and the iron powder reduction-crystallization method. The iron powder reduction-crystallization method had a higher iron removal efficiency than that of ammonium iron sulfate crystallization method. Considering the loss of liquid phase and the economics of industrialization of the method, the iron powder reduction-crystallization method was used for iron removal, and the effects of different iron amounts on the Fe^3+^ reduction as well as the formation of the third phase during the extraction process were studied systematically.

Different amounts of iron powder were explored on the crystallization, and the concentration changes of Fe^3+^ and Fe^2+^ in the solution are shown in Figure 3b–d. From these figures, it can be seen that, with the increase in the amount of iron powder, the amount of total iron tended to increase, but the amount of Fe^3+^ was reduced, while Fe^2+^ content was increased accordingly. When mFe:mFe^3+^ = 0.5, the concentration of Fe^3+^ in the solution decreased to 3.66 g/L, indicating a great reduction efficiency. The reduced leached solution was placed in a low-temperature thermostatic bath (0 °C) for crystallization, and Fe^2+^ was crystallized in the form of FeSO_4_·7H_2_O. The amount of Fe^2+^ in each group was dropped and finally floated at 33 g/L, indicating that the crystallization saturation of FeSO_4_·7H_2_O at 0 °C was reached and no more crystals can be precipitated. The amount of Fe^3+^ and Fe^2+^ did not change much after solvent extraction. Meanwhile, the content of all elements except Fe increased due to the sightly reduction in the liquid phase (Table 3).

### 2.4. Solvent Extraction of Scandium

#### 2.4.1. Effect of Iron Powder Reduction on Emulsification

**Figure 4:**
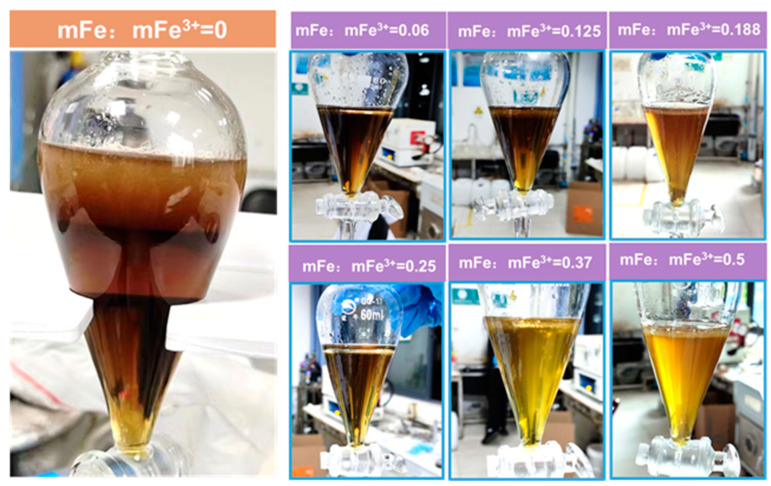
Generation of the third phase of the extraction process.

**Figure 5:**
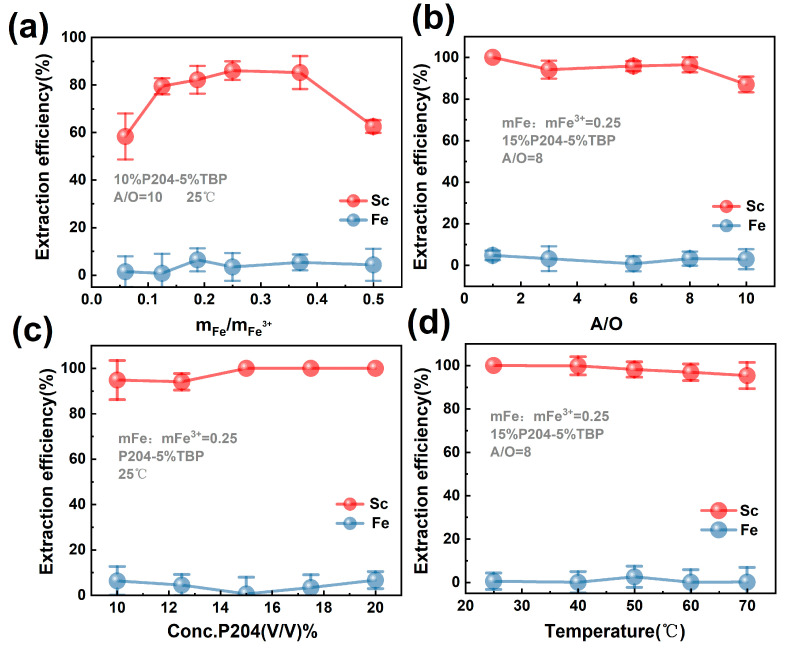
(**a**–**d**) show the effects of iron removal, A/O ratio, P204 concentration and temperature on the extraction effect, respectively.

The third phase generation during the extraction process after iron removal is shown in Figure 4 and the extraction results are depicted in Figure 5. According to Figure 4, it can be seen that the generation of the third phase was mitigated with the increase in the amount of iron powder. When mFe:mFe^3+^ = 0.25, the third phase was almost not being generated, and the oil phase became clarified. With the increasing amount of iron powder used, there was a generation of the third phase again during which even more Fe(III) was removed. This phenomenon may be due to the additional iron powder added, after which the consumption of H^+^ increased, leading to the rise of pH value and a third phase generated due to the hydrolysis of impurities.

As can be seen in Figure 5a, with the increase in mFe:mFe^3+^, the extraction efficiency of scandium reached the maximum value of 86% at mFe:mFe^3+^ = 0.25 from 58.33% at mFe:mFe^3+^ = 0.06, but after that the extraction efficiency decreased. When the solvent extraction was carried out at mFe:mFe^3+^ = 0.5, the increase in pH of the leachate and the generation of the third phase led to a decrease in the extraction efficiency of Sc. Also, the extraction of Fe was 3.39% at mFe:mFe^3+^ = 0.25. Therefore, to the optimal iron powder amount was mFe: mFe^3+^ = 0.25 during the iron removal process.

#### 2.4.2. Effect of A/O Ratio

The effect of A/O ratio on the extraction was investigated using the leached solution after iron removal as feedstock at the ratio of mFe:mFe^3+^ = 0.25. The extractant of 10% P204-5% TBP diluted with sulfonated kerosene was used during the extraction. The effect of A/O ratio on the extraction is shown in Figure 5b. With the increase in oil phase share, the extraction efficiencies of Sc and Fe showed an increasing trend. When A/O = 8, the extraction efficiency of Sc is 96.42%, after which the extraction efficiency almost reached equilibrium. When A/O = 1, the scandium extraction efficiency could reach 100%, and the iron extraction efficiency also reached the highest value, 4.7%. The subsequent extraction conditions were carried out at A/O = 8.

#### 2.4.3. Effect of P204 Concentration

The effect of P204 concentration on the extraction efficiency is shown in Figure 5c. With the increase in P204 concentration, the extraction efficiency of scandium increased and plateaued. The extraction efficiency was close to 100% when its concentration was higher than 15%. Meanwhile, with the increase in P204 concentration, the extraction efficiency of Fe varied. When the concentration of P204 was 15%, the extraction efficiency of Fe was 0.53%. Therefore, 15% P204-5% TBP was selected for the experiments. In the system of P204-TBP, P204(HA) existed in the form of dimer (H_2_A_2_), which was the main loading organic phase of Sc. Since the coordination number of Sc^3+^ was two times of its valence, the formed chelate Sc(HA_2_)_3_ was not saturated. The presence of TBP allowed the formation of the chelate Sc(HA_2_)_3_·TBP or Sc(HA_2_)_3_·2TBP, which can improve the distribution ratio, thus improving the extraction efficiency and selectivity of Sc. Therefore, the extraction system was more effective when the concentration ratio of P204:TBP was about 3:1 [2]. And further increase in P204 content, the organic phase that can load more Sc^3+^, enhancing the extraction efficiency. However, the organic phase can also be loaded by Fe^3+^, increasing the extraction of Fe^3+^.

#### 2.4.4. Effect of Temperature

The extraction system of 15% P204-5% TBP was used to extract the leached solution after iron removal at A/O = 8 to investigate the effect of temperature on the extraction. The effect of temperature on the extraction is shown in Figure 5d. With the increase in temperature, the extraction efficiency of Sc showed a slight decreasing trend, and the extraction efficiency of Fe did not change much. Therefore, it was chosen to carry out the extraction experiments at 25 °C.

**Figure 6:**
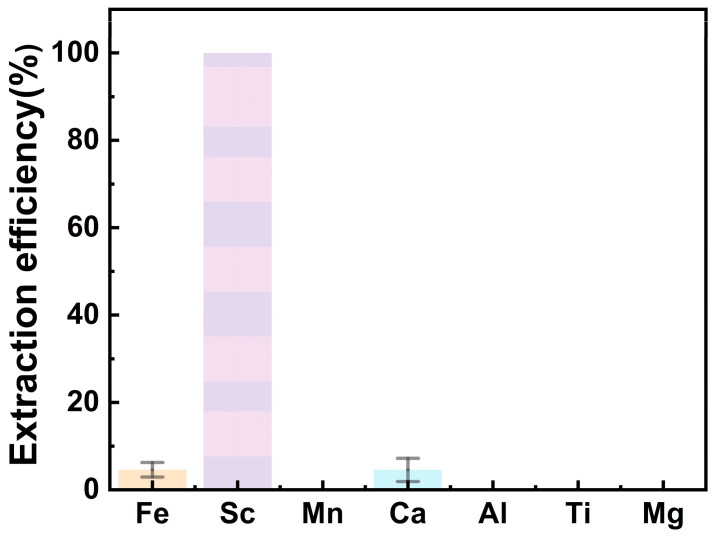
Comparison of the extraction of each ion under the optimal process conditions.

Based on the above results, the optimum process conditions for extraction were obtained as pre-Fe(III) removal withmFe:mFe^3+^ = 0.25, and a extractants of 15% P204-5% TBP, A/O = 8, at 25 °C. The extraction of other impurity elements under these extraction conditions is shown in Figure 6. It can be seen that the extraction efficiency of Sc can reach 100%, while the extraction efficiencies of Fe and Ca were very low with 4.58% and 4.56%, respectively. The elements of Mn, Mg, Al and Ti were hardly extracted. The contents of each element in the raffinate can be seen in Table 3.

### 2.5. Conceptual Flowchart for the Recovery of Scandium from Scandium-Containing Waste Acid and Slag

**Figure 7:**
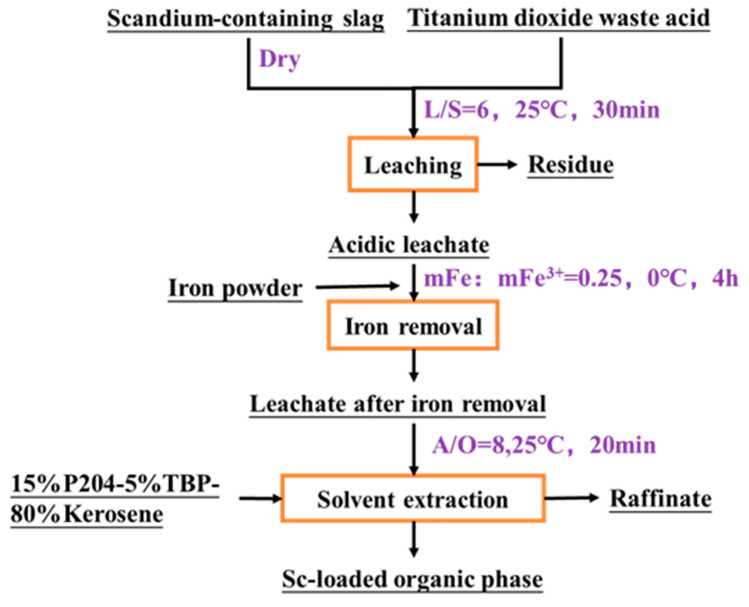
Conceptual flow diagram for the recovery of scandium.

According to the above results, a route for recovering scandium from titanium dioxide waste acid and scandium-containing waste slag was proposed. It is shown in Figure 7. Firstly, two wastes were leached synergistically to enrich scandium. Under the optimal leaching conditions, i.e., liquid-solid ratio of 5, temperature of 25 °C, leaching time of 30 min, the leaching efficiency of Sc can reach 87.72% and the content of Sc in the leached solution was enriched from 15.1 mg/L to 38.1 mg/L. However, the content of Fe in the leached solution was also enriched. To reduce the effect of Fe^3+^ i on the solvent extraction process, Fe^3+^ in the leachate was removed by Fe powder reduction followed by crystallization method. A iron powder amount of mFe:mFe^3+^ = 0.25 was added, and the Fe^3+^ was removed by crystallization in the form of FeSO_4_·7H_2_O. By this means, the extraction efficiency of Sc was improved, and the emulsification phenomenon was mitigated in the solvent extraction process. The Fe(III)-depleted leached solution was extracted with 15% P204-5% TBP, and the Sc could be completely extracted at room temperature with A/O = 8 for 20 min. The comparison of the extraction process flow with that of other studies is presented in Table 4. Compared with other processes, this process was carried out to remove Fe^3+^ before extraction, which reduced the formation of third phase caused by Fe^3+^ and improved the selectivity and extraction efficiency of Sc. The study provided a new reference process for scandium recovery. However, due to the large dosage of P204, the economic efficiency of a single extraction is relatively low. In the subsequent stages, P204-TBP can be utilized for secondary or multiple-stage extraction to further recover scandium, thereby increasing the concentration of scandium in the organic phase and achieving a more economically efficient outcome.

## 3. Materials and Methods

### 3.1. Reagents

In this study, titanium dioxide waste acid was provided by Chongqing Titanium Industry Co., Ltd. of Pangang Group (Chongqing, China), and scandium-containing waste slag was provided by Panzhihua Iron and Steel Company (Panzhihua, Chian). The waste slag was ground to less than 100 mesh before use. The organic solvents P204, TBP and other extraction solvents were provided by Chongqing Kangpu Chemical Industry (Chongqing, China).

### 3.2. Experiments

In the leaching experiments, the scandium-containing slag was first ground to below 100 mesh and dried in an oven at 80 °C for 24 h. The slag was leached by titanium dioxide waste acid at temperature range of 25–70 °C for30–90 min with L/S (the ratio of the liquid phase to the solid phase) of 6–12. After leaching, filtration was carried out, and the concentration of metal ions in the leached solution was determined using inductively coupled plasma atomic emission spectrometry (ICP-OES, Optima 8300DV, PE Inc., Cincinnati, OH, USA). The leaching efficiency (I%) of metal ions was calculated according to Equation (1), and the effects of temperature (25 °C, 40 °C, 50 °C, 60 °C, 70 °C), liquid-solid ratio (6, 8, 9, 10, 12) and time (30 min, 45 min, 60 min, 75 min, 90 min) on the leaching efficiency were explored.

(1) $$I=\frac{C_{i}\times V_{i}}{C_{RL}\times V_{RL}+M_{RS}\times \omega_{i}}\times 100\%$$

where C_i_ is the concentration of metal elements in the leach solution (g/L); V_i_ is the volume of leach solution used for testing (L); C_RL_ is the concentration of metal elements in the spent acid (g/L); V_RL_ is the volume of metal elements in the spent acid used for testing (L); M_RS_ is the mass of the spent slag used for leaching (g) and ω_i_ is the percentage of metal elements in the slag (%).

This study compared four methods for iron removal, i.e., 1-potassium ferricyanide method, 2-jarosite process, 3-ferric amine sulfate crystallization method, and 4-iron powder reduction-crystallization method. Potassium ferricyanide method (Equation (2)) experiment was carried out at 55 °C with the ratio of potassium ferricyanide: leached solution = 200 g/L. After heating and stirring for 1 h, the solution was kept warm for 30 min and then pumped and filtered to obtain the iron removal solution. Jarosite process (Equations (3) and (4)) is a very commonly used method of iron removal. 50 mL leached solution was heated to 90 °C, and then added NH_3_-H_2_O was dropped. The jarosite was formed and filtrated to obtain the iron removal solution. Ammonium iron sulfate crystallization method (Equations (5) and (6)) was carried out by adding (NH_4_)_2_SO_4_ to generate ammonium iron sulfate with lower solubility, thus achieving the iron removal. In detail, 1.8 g of (NH_4_)_2_SO_4_ was added to 20 mL leached solution, and the mixture was stirred at 25 °C and equilibrated for 60 min. After that, the mixture was filtered to obtain the iron removal solution. The iron powder reduction-crystallization method (Equation (7)) used the addition of iron powder to reduce ferric ions to ferrous ions, and finally crystal as FeSO_4_·7H_2_O, thus achieving the iron removal. According to the Fe^3+^ content in the leaching solution, iron powder was added in a certain proportion (mFe:mFe^3+^ = 0.06, 0.125, 0.188, 0.25, 0.37, 0.5). The leached solution was placed in an oil bath at 50 °C and stirred until the reaction of iron powder was complete. The reduced solution was then placed in a low-temperature thermostatic bath (provided by Changzhou Noda Instrument Technology Co., Ltd., Changzhou, China), crystallized at 0 °C for 4 h and then filtered, to obtain the iron removal solution. The changes of iron and scandium in the leached solution and the iron removal solution were determined by ICP-OES method. Meanwhile, for the iron powder reduction-crystallization method, potassium dichromate titration (Equation (8)) was used to determine the content of ferrous iron ions in the solution, so as to obtain the ratio of ferrous to ferric ions. The reaction equations and calculations involved are shown in (2)–(10):

(2) $${Fe}^{3+}+{\left[Fe{\left(CN\right)}_{6}\right]}^{4-}\to {Fe}^{2+}+{\left[Fe{\left(CN\right)}_{6}\right]}^{3-}$$

(3) $$3Fe_{2}(SO_{4}{)}_{3}+10H_{2}O+NH_{4}OH=(NH_{4}{)}_{2}Fe_{6}(SO_{4}{)}_{4}(OH{)}_{12}↓+5H_{2}SO_{4}$$

(4) $$3Fe_{2}(SO_{4}{)}_{3}+12H_{2}O+Na_{2}SO_{4}=Na_{2}Fe_{6}(SO_{4}{)}_{4}(OH{)}_{12}↓+6H_{2}SO_{4}$$

(5) $${Fe}^{3+}+{\left({NH}_{4}\right)}_{2}SO_{4}\to {NH}_{4}Fe(SO_{4}{)}_{2}$$

(6) $${Fe}^{2+}+{\left({NH}_{4}\right)}_{2}SO_{4}\to {\left({NH}_{4}\right)}_{2}Fe(SO_{4}{)}_{2}$$

(7) $$Fe+2{Fe}^{3+}=3{Fe}^{2+}$$

(8) $$6{Fe}^{2+}+{{Cr}_{2}O_{7}}^{2-}+14H^{+}\to 6{Fe}^{3+}+2{Cr}^{3+}+7H_{2}O$$

(9) $$M_{{Fe}^{2+}}=\frac{C\times V\times 6\times 56}{V_{l}}$$

(10) $$M_{{Fe}^{3+}}=M_{Fe}-M_{{Fe}^{2+}}$$

where C is the concentration of potassium dichromate titrant used (mol/L); V is the volume of potassium dichromate titrant consumed (mL) and V_l_ is the volume of leachate taken (mL).

Solvent extraction of the scandium-containing leached solution was carried out using the co-extraction system of P204-TBP. The leached solution, P204, TBP and sulfonated kerosene were added into a beaker and placed in a water or oil bath for stirring extraction. After that, the solution was poured into a separating funnel for oil-water separation, the aqueous phase was taken for ICP-OES test, the scandium extraction (E, %) was calculated according to Equation (11), and the effects of the amount of iron powder (mFe:mFe^3+^ = 0.06, 0.125, 0.188, 0.25, 0.31, 0.37, 0.5), A/O ratio (1, 3, 6, 8, 10), P204 content (10%, 12.5%, 15%, 17.5%, 20%) and temperature (25 °C, 40 °C, 50 °C, 60 °C, 70 °C) were investigated.

(11) $$E=\frac{C_{L}-C_{OL}}{C_{L}}\times 100\%$$

where C_L_ is the concentration of the metal in the iron removal solution (g/L) and C_OL_ is the concentration of the metal element in the aqueous phase after extraction (g/L).

### 3.3. Sample Characterization

X-ray diffractometer (D2-Phaser, Bruker, Bremen, Germany) was used to determine the mineralogical composition of raw materials and mineralization products. X-ray fluorescence spectrometer (S8 Tiger, Bruker, Germany) was used to analyze the chemical composition of raw materials. Inductively coupled plasma atomic emission spectrometry (Optima 8300DV, PE Inc., USA) was a technique used for elemental analysis that allows for the simultaneous measurement of the concentrations of multiple elements.

## 4. Conclusions

In this study, titanium dioxide waste acid was used to leach scandium-containing waste slag, and the co-extraction system of P204-TBP was used to extract Sc from the leached solution after iron removal. The optimal process conditions were determined. The results showed that the leaching conditions of liquid-solid ratio L/S = 6, 25 °C and 30 min were the most suitable for the leaching of Sc, under which the leaching efficiency of Sc could reach 87.72%, while other impurity metals were lower in comparison. Four iron removal methods were compared, among which the iron powder reduction-crystallization method was the most suitable. At the ratio of mFe:mFe^3+^ = 0.25, the problem of the generation of the third phase in the extraction process can be solved. Under the extraction conditions of 15% P204-5% TBP, A/O of 8 and extraction time of 20 min, the extraction efficiency of Sc could reach 100%, while the extraction efficiencies of Fe and Ca were as low as 4.58% and 4.56%, and Mn, Al, Ti and other elements were hardly extracted.

## Figures and Tables

**Table 1:** X-ray fluorescence (XRF) test results of waste slag.

Element	Fe	Mn	Ti	Al	Mg	Zr	Ca	Sc
Content (wt%)	47.20	5.42	0.50	0.93	8.08	0.02	3.36	n/a

**Table 2:** ICP-OES test results of waste slag and titanium dioxide waste acid.

Element	Sc	Ti	Fe	Mn	Ca	Al	Mg
Waste slag (wt%)	0.017	0.556	29.3	2.57	4.77	0.616	8.72
Titanium dioxide waste acid (g/L)	0.0151	2.65	46.43	1.71	3.89	2.95	5.36

**Table 3:** Changes in the content of scandium and other impurity elements.

Element Concentration (mg/L)	Fe	Sc	Ca	Mn	Ti	Al	Mg
Titanium dioxide waste acid	46,430	15.1	3890	1710	2650	2950	5359.6
Leachate	80,393	38.1	277	6046.9	2536.8	4755.6	15,144.3
Leachate after iron removal	64,716.1	40.3	283.0	6221.3	2562.2	4774.8	15,206.6
Solvent extraction raffinate	61,752.7	0	270.1	6228.8	2563.7	4783.5	15,793.7

**Table 4:** Comparison of the extraction process described in this paper with other research processes.

Extractant	Acid	Conditions	Rate (%)	Refs.
3%P204	H_2_SO_4_	A/O = 5, 40 °C	99.5	[28]
10% P204-5% TBP	Titanium white waste acid	A/O = 30, 30 min, 25 °C	99	[9]
8% P204-2% TBP	Titanium white waste acid	A/O = 10, 25 °C, 20 min	99.98	[29]
10% P204-5% TBP	7 mol/LH_2_SO_4_	A/O = 10, 25 °C, 30 min	99.90	[30]
10%P204-5%TBP	H_2_SO_4_	A/O = 10, 30 min	>99	[31]
15%P204-5%TBP	Titanium dioxide waste acid	A/O = 8, 25 °C, 20 min	100	This work

## Data Availability

The data presented in this study are available on request from the corresponding authors.
